# Molecular Epidemiology of Classic, MLB, and VA Astroviruses in Children with Acute Gastroenteritis, 2014–2021: Emergence of MLB3 Strain in Japan

**DOI:** 10.1128/spectrum.00700-23

**Published:** 2023-05-04

**Authors:** Shoko Okitsu, Pattara Khamrin, Toshiyuki Hikita, Yuko Shimizu-Onda, Aksara Thongprachum, Satoshi Hayakawa, Niwat Maneekarn, Hiroshi Ushijima

**Affiliations:** a Division of Microbiology, Department of Pathology and Microbiology, Nihon University School of Medicine, Tokyo, Japan; b Department of Microbiology, Faculty of Medicine, Chiang Mai University, Chiang Mai, Thailand; c Center of Excellence in Emerging and Re-emerging Diarrheal Viruses, Chiang Mai University, Chiang Mai, Thailand; d Hikita Pediatric Clinic, Gunma, Japan; e Faculty of Public Health, Chiang Mai University, Chiang Mai, Thailand; Changchun Veterinary Research Institute

**Keywords:** astrovirus, Japan, children, acute gastroenteritis, epidemiology

## Abstract

Human astroviruses (HAstVs) are important causative pathogens of acute gastroenteritis (AGE) in children worldwide. MLB and VA HAstVs, which are genetically distinct from the previously known classic HAstVs, have been detected since 2008. To investigate the role of HAstVs in AGE, we conducted molecular detection and characterization of HAstVs circulating in children with AGE in Japan from 2014 to 2021. Out of 2,841 stool samples, HAstVs were detected in 130 (4.6%). MLB1 was the predominant genotype detected (45.4%), followed by HAstV1 (39.2%), MLB2 (7.4%), VA2 (3.1%), HAstV3 (2.3%), HAstV4, HAstV5, and MLB3 (0.8% each). The results demonstrated that HAstV infection in pediatric patients in Japan was dominated by the two major genotypes MLB1 and HAstV1, with a small proportion of other genotypes. The overall infection rates of MLB and VA HAstVs were higher than those of classic HAstVs. The HAstV1 strains detected in this study belonged solely to lineage 1a. The rare MLB3 genotype was detected for the first time in Japan. All three HAstV3 strains belonged to lineage 3c based on the ORF2 nucleotide sequence and were shown to be recombinant strains.

**IMPORTANCE** HAstVs are one of the pathogens of viral AGE and are considered the third most common viral agents of AGE after rotavirus and norovirus. HAstVs are also suspected to be the causative agents of encephalitis or meningitis in immunocompromised patients and elderly persons. However, little is known about the epidemiology of HAstVs in Japan, especially that of MLBs and VA HAstVs. This study demonstrated epidemiological features and molecular characterization of human astroviruses encompassing a 7-year study period in Japan. This study highlights the genetic diversity of HAstV circulating in pediatric patients with acute AGE in Japan.

## INTRODUCTION

Human astroviruses (HAstVs) are one of the leading causative agents of acute gastroenteritis (AGE), especially in infants and children, elderly persons, and immunocompromised patients. Since the implementation of rotavirus vaccines has led to a decrease in rotavirus infection, HAstV has become a more important diarrhea-causing pathogen, following norovirus and sapovirus (1). HAstV infection may result in a wide range of symptoms from asymptomatic to mild watery diarrhea, and systemic infection of the central nervous system in association with meningitis and encephalitis has also been reported (2–4). The classic HAstV was first discovered in 1975 in stool samples of children with AGE (5), whereas the MLB and VA HAstVs were detected in 2008 (6) and 2009 (7), respectively. Currently, several genotypes of classic HAstV (HAstV1 to -V8), MLB HAstV (MLB1 to -3), and VA HAstV (VA1 to -5) have been identified (2). Astroviruses are small, nonenveloped viruses classified in the genus *Mamastrovirus* of the family *Astroviridae*. Astroviruses possess a single-stranded, positive-sense RNA genome that encodes three open reading frames (ORFs): ORF1a and ORF1b, encoding nonstructural viral protease and polymerase, respectively; and ORF2, encoding structural capsid protein (8, 9). Epidemiological studies of classic HAstV infection in children with AGE in Asian, North and South American, European, and African countries have revealed that the prevalence of classic HAstV infection ranges from 0 to >20% (2, 10, 11). On the other hand, the MLB and VA HAstVs have also been detected worldwide, at a prevalence lower than that of classic HAstV (2, 10). In Japan, the prevalence of classic HAstV infection in children with AGE ranges from 1.6% to 5.2% (12–16); however, knowledge of the epidemiology of MLB and VA HAstVs is limited. So far, only one study has reported that the prevalence of MLB HAstV was higher than that of the classic HAstV (16). The aim of the present study was to investigate the prevalence and genotype distribution of classic HAstV and MLB and VA HAstVs in children with AGE in Japan during the study period of 2014 to 2021.

## RESULTS

### Prevalence, age distribution, and seasonality of HAstV infection.

**FIG 1:**
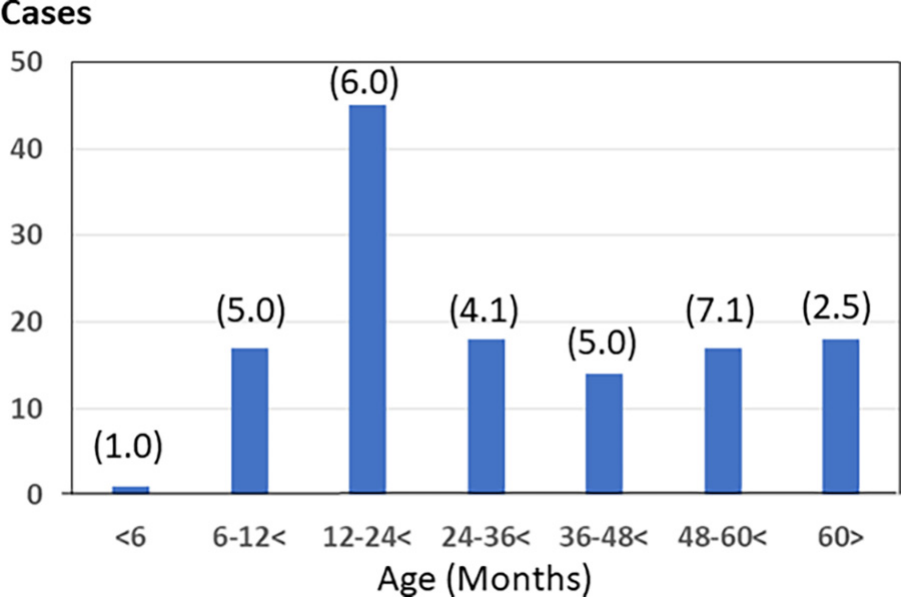
Distribution of human astrovirus (HAstV) in pediatric patients with different age groups. Numbers in parentheses indicate the percentage of positive cases.

Stool samples were collected from children with AGE who visited an outpatient clinic in Gunma Prefecture, Japan, from July 2014 to June 2021. The 1-year periods designated in this study ran from July to June of the following year. The patients included in this study were diagnosed with viral-induced AGE by the physician based on clinical symptoms, including watery diarrhea, vomiting, and fever. Stool samples from patients aged more than 180 months old were excluded. A total of 2,841 stool samples were obtained, and the ratio of male to female subjects was 1,502:1,339. During the 7-year study period, HAstV was detected in 130 out of 2,841 (4.6%) stool samples collected from children with AGE by amplifying and sequencing the nucleotide sequences of the partial RNA-dependent RNA polymerase (RdRp) region (Table 1). The HAstV detection rates for each year varied from 1.8% in the period of 2014 to 2015 to 7.1% in 2016 to 2017. The detection rates were 5.1% in 2015 to 2016, 2.9% in 2017 to 2018, and 3.3% in both 2018 to 2019 and 2019 to 2020. It should be noted that very few samples were collected from 2020 to 2021, and no HAstV was detected. The highest number of HAstV infections was observed in children 12 to <24 months old, and the highest detection rate of 7.1% was observed in children 48 to <60 months old, followed 6.0% in those aged 12 to <24 months old (Fig. 1). The ratio of male to female HAstV-positive patients was 73:57. The HAstV infection rate was not significantly different between males and females (chi-square test: *P* = 0.46).

**TABLE 1:** Prevalence and genotype distribution of HAstV in Japanese children with AGE, 2014 to 2021^*a*^

Study period	Samples tested, *n*	HAstV positive, *n* (%)	HAstV genotypes, *n* (%)^*b*^							
			HAstV1	HAstV3	HAstV4	HAstV5	MLB1	MLB2	MLB3	VA2^*c*^
2014–2015	553	10 (1.8)	1	0	0	0	6	0	0	3
2015–2016	866	44 (5.1)	11	3	1	0	18	10	0	1
2016–2017	836	59 (7.1)	29	0	0	0	30	0	0	0
2017–2018	273	8 (2.9)	3	0	0	1	4	0	0	0
2018–2019	181	6 (3.3)	6	0	0	0	0	0	0	0
2019–2020	90	3 (3.3)	1	0	0	0	1	0	1	0
2020–2021	42	0	0	0	0	0	0	0	0	0
Total	2,841	130 (4.6)	51 (39.2)	3 (2.3)	1 (0.8)	1 (0.8)	59 (45.4)	10 (7.4)	1 (0.8)	4 (3.1)

a HAstV, human astrovirus; AGE, acute gastroenteritis.

b Total percentage is not 100% because the data are rounded.

c The nucleotide sequences of partial capsid regions of two VA2 strains could not be amplified; therefore, the genotypes of these strains were determined by screening the nucleotide sequences of partial RdRp regions.

**FIG 2:**
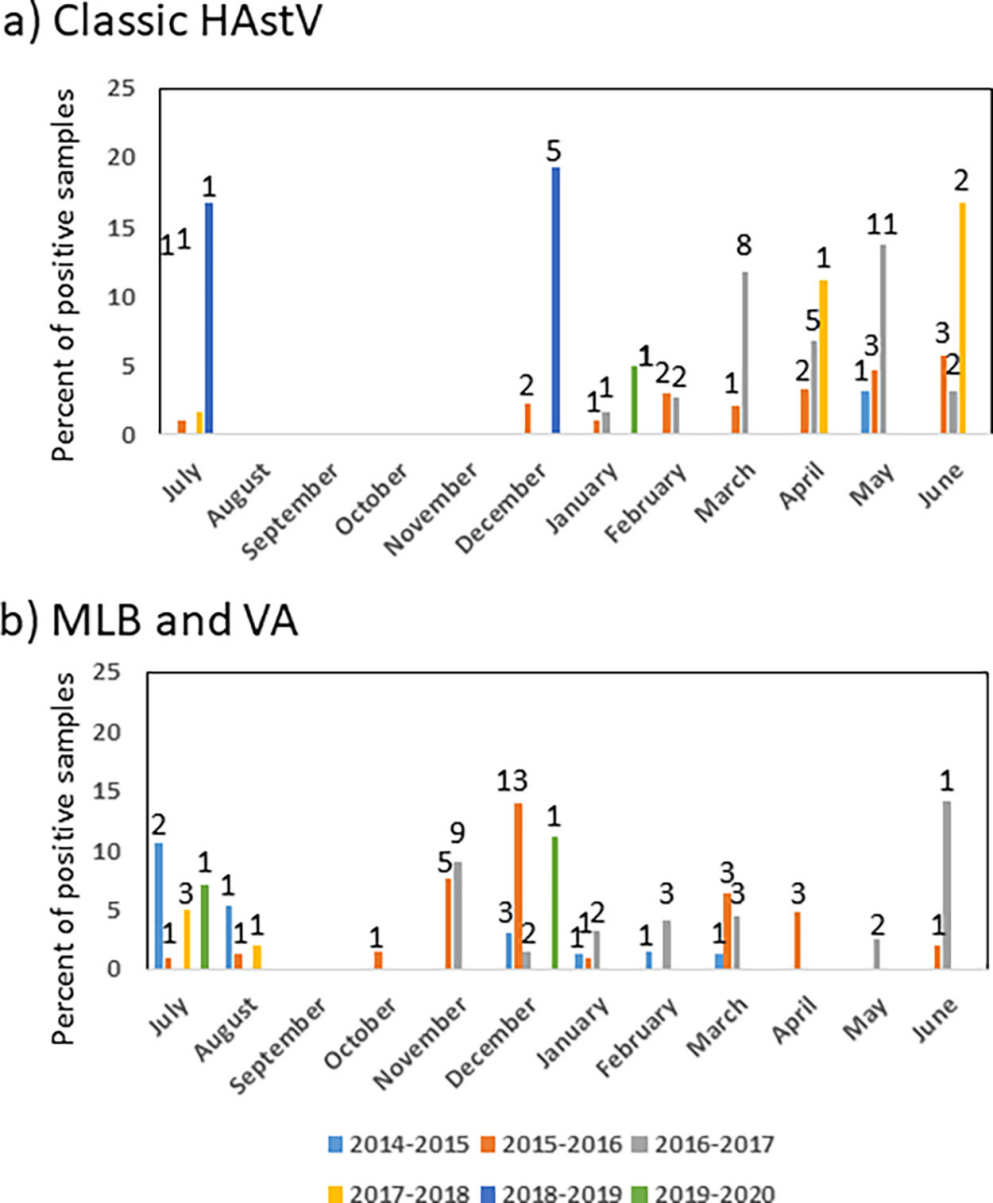
Monthly distribution of HAstV. Study year periods ran from July to June of the following year. (a) Classic HAstV. (b) MLB and VA HAstVs. Numbers indicate percentage of positive cases.

Classic HAstV infection was detected during the cool and dry months in Japan from December to April, and in the hot and wet months from May to July. However, HAstV infection was not detected during the period of August to November. On the other hand, the MLB and VA HAstVs were detected year-round except for September (Fig. 2).

### Distribution of HAstV genotypes.

HAstV genotypes were assigned based on the nucleotide sequences of the capsid regions. The nucleotide sequences, including RdRp-capsid junction regions of all classic HAstV and MLB strains, were successfully amplified and sequenced. However, we could only amplify the RdRp-capsid junction regions from two of the four VA2 strains detected in this study; therefore, the genotypes of these two VA2 strains were assigned based on partial RdRp nucleotide sequences. As shown in Table 1, MLB1 was the most prevalent genotype (45.4%), followed by HAstV1 (39.2%), MLB2 (7.4%), VA2 (3.1%), HAstV3 (2.3%), HAstV4, HAstV5, and MLB3 (0.8% each). MLB1 (45.4%) and HAstV1 (39.2%) were the co-predominant genotypes detected in this study, whereas other HAstV genotypes, including HAstV3, -V4, and -V5 and MLB2, MLB3, and VA2 were detected at much lower rates. It should be noted that a rare MLB3 strain, JP18634, was detected for the first time in Japan in this study. Interestingly, the MLB2 strains were detected only from 2015 to 2016 and the VA2 strain was not detected after 2016.

### Prevalence of mono and mixed infections of HAstV.

An analysis of HAstV genotype distribution in AGE pediatric patients with mono-HAstV infection and mixed infections of HAstV together with other diarrheal viruses revealed that 68.5% of HAstV-positive cases were mixed infections of HAstV and other enteric viruses, including rotavirus A, norovirus GII, adenovirus, sapovirus, parechovirus A, and enterovirus (Table 2). The prevalence of mono infection of classic HAstV was the highest (39.3%), followed by MLB (25.7%) and VA (25.0%). Norovirus GII was the predominant virus detected among the enteric viruses co-infecting with HAstV, followed by rotavirus A. In addition, one patient infected with MLB3 HAstV was also co-infected with norovirus GII. However, the detection of bacterial pathogens, toxins, and parasites that might be causative agents of AGE was not investigated in this study.

**TABLE 2:** Prevalence of mono and mixed infections of HAstV and other diarrheal viruses^*a*^

HAstV	Infection type, no. of cases (%)		No. and patterns of HAstV mixed infection						
	Mono	Mixed	RVA	NVGII	AdV	SaV	PeVA	EV	≥2 viruses
Classic	22 (39.3)	34 (60.7)							
V1	22	29	15	6	1		1		6^*b*^
V3	0	3	2	1					
V4	0	1		1					
V5	0	1		1					
MLB	18 (25.7)	52 (74.3)							
1	13	46	5	29	1	2	1	1	7^*c*^
2	5	5		2					3^*d*^
3	0	1		1					
VA	1 (25.0)	3 (75.0)							
VA2	1	3	1						2^*e*^
Total	41 (31.5)	89 (68.5)	23	41	2	2	2	1	18

a HAstV, human astrovirus; RVA, rotavirus A; NVGII, norovirus genogroup II; AdV, adenovirus; SaV, sapovirus; PeVA, parechovirus A; EV, enterovirus.

b RVA + NVGII (*n* = 4), NVGII + SaV (*n* = 1), and NVGII + EV (*n* = 1).

c RVA + NVGII (*n* = 3), RVA + EV (*n* = 1), NVGII + PeVA (*n* = 2), and NVGII + AdV + PeVA (*n* = 1).

d NVGII + EV (*n* = 3).

e RVA + NVGII (*n* = 2).

### Phylogenetic analysis of nucleotide sequences of the partial capsid region of classic HAstV strains.

**FIG 3:**
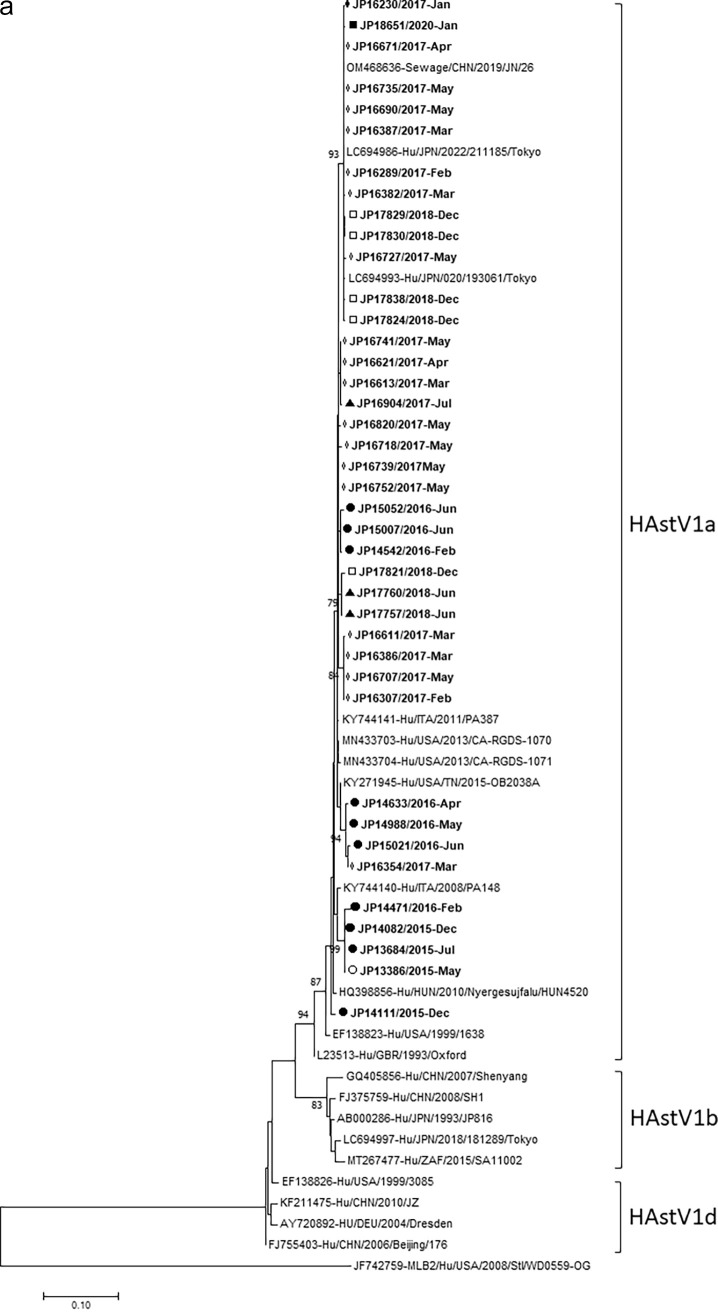

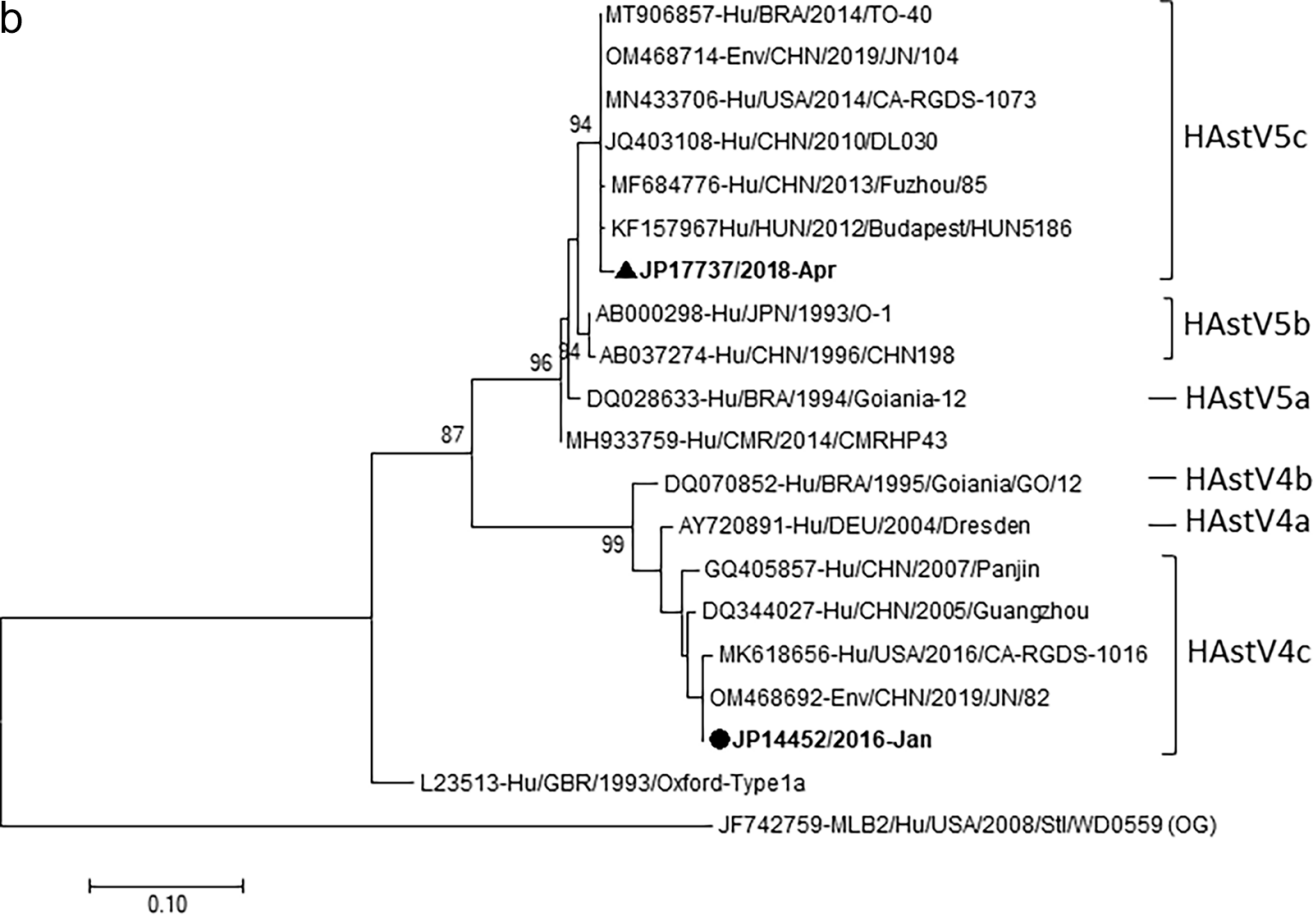
Phylogenetic analysis of nucleotide sequences of the partial capsid region of classic HAstV. (a) Classic HAstV1 (510 bp); (b) classic HAstV4 and HAstV5 strains (319 bp). Trees were constructed using the maximum-likelihood method based on the Kimura two-parameter model in panel a and the Tamura three-parameter model in panel b. Scale bar shows nucleotide substitutions per site and bootstrap values of >75 are indicated. The HAstV strains detected in this study are shown in bold type. Symbols indicate strain names and year of collection: empty circle (○), 2014 to 2015; filled circle (●). 2015 to 2016; empty diamond (◊), 2016 to 2017; filled triangle (▴), 2014 to 2018; empty square (□), 2018 to 2019; filled square (■), 2019 to 2020. The JF742759-MLB2/Hu/USA/2008/Stl/WD0559 strain is used as an outgroup. Hu, human; Env, environmental water (sewage).

HAstV1, -V4, and -V5 genotypes were further characterized for their lineages by phylogenetic analyses of the partial capsid nucleotide sequences. The representative HAstV1 strains detected in this study were compared with the reference strains of lineages, 1a, 1b, and 1d. It was observed that all the HAstV1 strains detected in this study belonged to lineage 1a, as shown in Fig. 3a. The forty strains of HAstV1 detected in this study, which were collected in different years from 2015 to 2020, were relatively similar to each other, with nucleotide sequence identities ranging from 97.2% to 100%. They were closely related to the reference strains reported previously from Japan (LC694986, LC694993), China (OM468636), the United States (MN433703, MN433704, and KY271945), and Italy (KY744141), with nucleotide sequence identities ranging from 97.0% to 100%. One each of the HAstV4 (JP14452) and HAstV5 (JP17737) strains detected in this study belonged to lineages 4c and 5c, respectively, as shown in Fig. 3b. The JP14452 strain was closely related to the classic HAstV4c reference strains previously reported from China (GQ405857, OM468692, and DQ344027) and the United States (MK618656), with nucleotide sequence identities ranging from 97.8% to 100%. The JP17737 strain was most closely related to the classic HAstV5c reference strains previously reported from China (MF684776, OM468714, and JQ403108), the United States (MN433706), Hungary (KF157967), and Brazil (MT906857), with nucleotide sequence identities ranging from 98.7% to 99.0%.

### Phylogenetic analyses of full-length genomes of HAstV3 strains.

**FIG 4:**
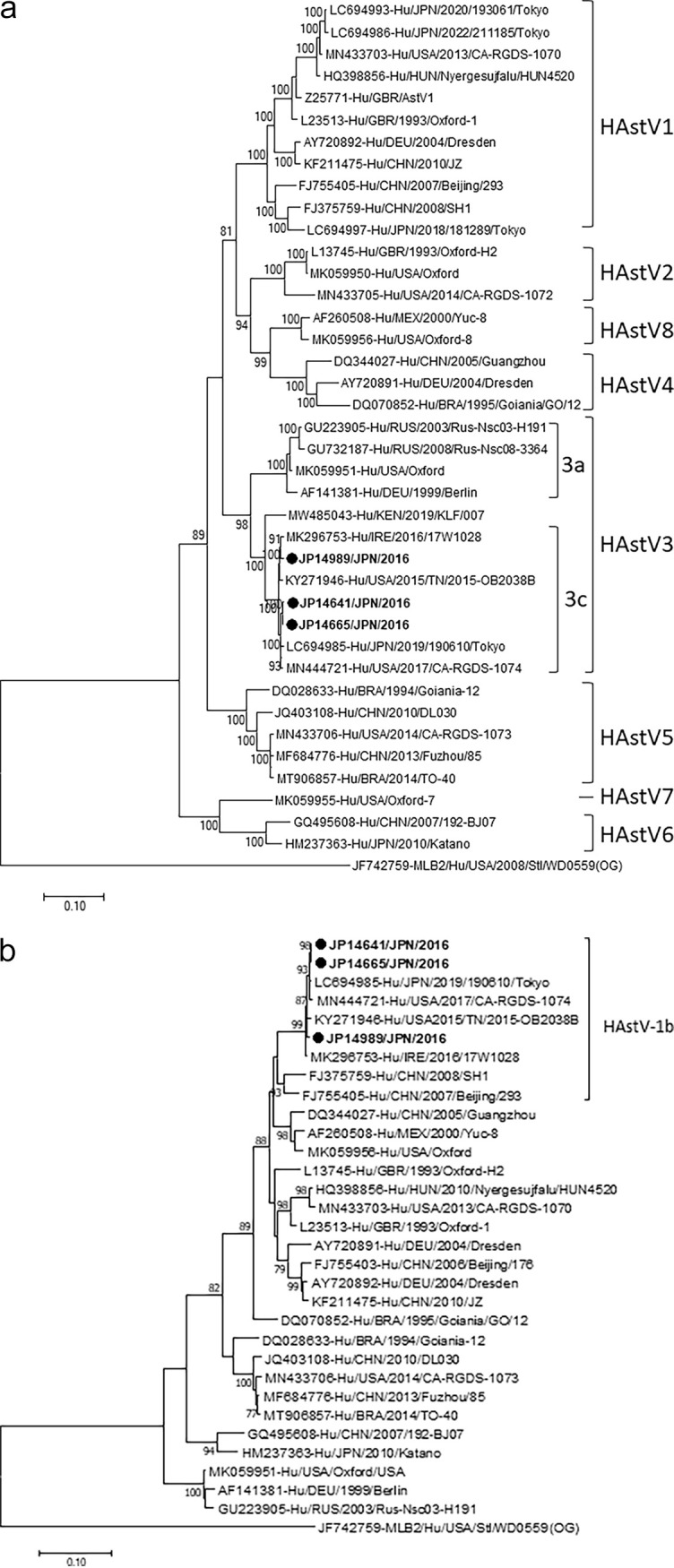

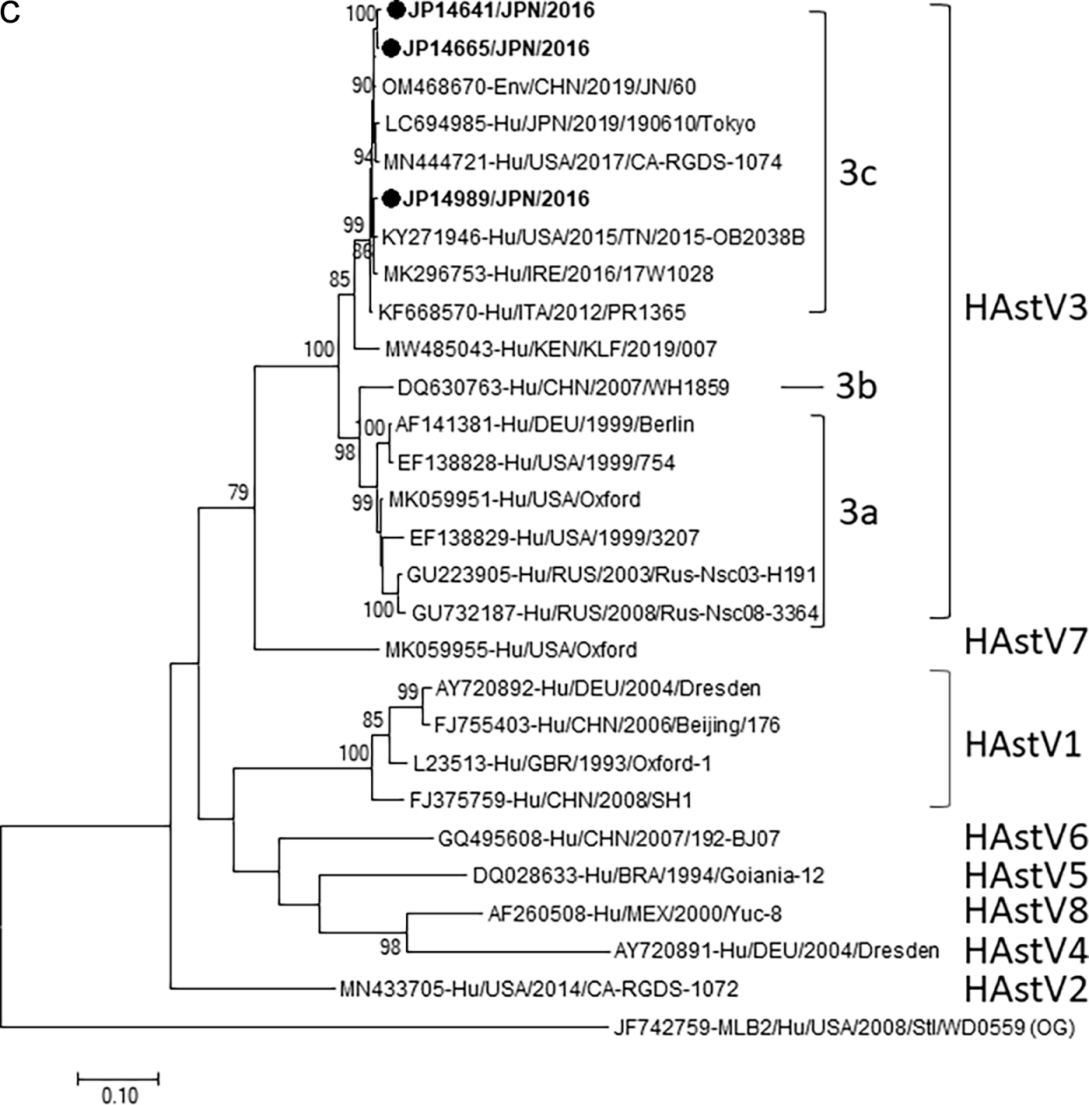
Phylogenetic analysis of the nucleotide sequences of HAstV3 strains detected in this study. (a) Full-genome sequences (6,411 bp), (b) ORF1b (1,473 bp), and (c) ORF2 (2,242 bp). Trees were constructed using the maximum-likelihood method based on the Tamura three-parameter model. Scale bar shows nucleotide substitutions per site and bootstrap values of >75 are indicated. HAstV strains detected in this study are shown in bold type. Lineages with strains names are designated based on the nucleotide sequences of ORF2. The JF742759-MLB2/Hu/USA/2008/Stl/WD0559 strain is used as an outgroup.

The full-length nucleotide sequences of the genomes of three HAstV3 strains (JP14641, JP14665, and JP14989) detected in this study were determined and compared with those of previously reported classic HAstV strains available in GenBank, as shown in Fig. 4a. The phylogenetic tree showed that the HAstV3 strains detected in this study formed a monophyletic branch with four other HAstV3c strains detected in Ireland (MK296753), the United States (KY271946 and MN444721), and Japan (LC694985), with nucleotide sequence identities ranging from 98.2% to 99.7%, suggesting that they belonged to the HAstV3c genotype. Comparison of the nucleotide sequences of our HAstV3 strains with those of the four reference strains of genotype 3a revealed that the nucleotide sequence identities were much lower, ranging from only 88.1% to 89.1%, suggesting that they did not belong to the 3a genotype. As shown in Fig. 4b, it was interesting to observe that the phylogenetic tree of ORF1b showed that the HAstV3 strains (JP14641, JP14665, and JP14989) detected in this study clustered together with the HAstV1b reference strain (FJ755405-Hu/CHN/2007/Beijing/293), with nucleotide sequence identities ranging from 93.0% to 93.6%, suggesting that the ORF1b of these strains belonged to the HAstV1b genotype. The phylogenetic tree of ORF2 nucleotide sequences of HAstV3 strains, shown in Fig. 4c, revealed that the ORF2 HAstV3 of the three strains detected in this study were closely related to those of the six HAstV3c reference strains, with nucleotide sequence identities ranging from 97.9% to 99.4%. Comparison of the ORF2 nucleotide sequence of our HAstV strains with that of the ORF2 of the KF668570-ITA/2012/PR1365 strain, which was first classified as the 3c genotype, ranged from 98.1% to 98.7%.

### Phylogenetic analysis of the full-length genome of MLB3.

**FIG 5:**
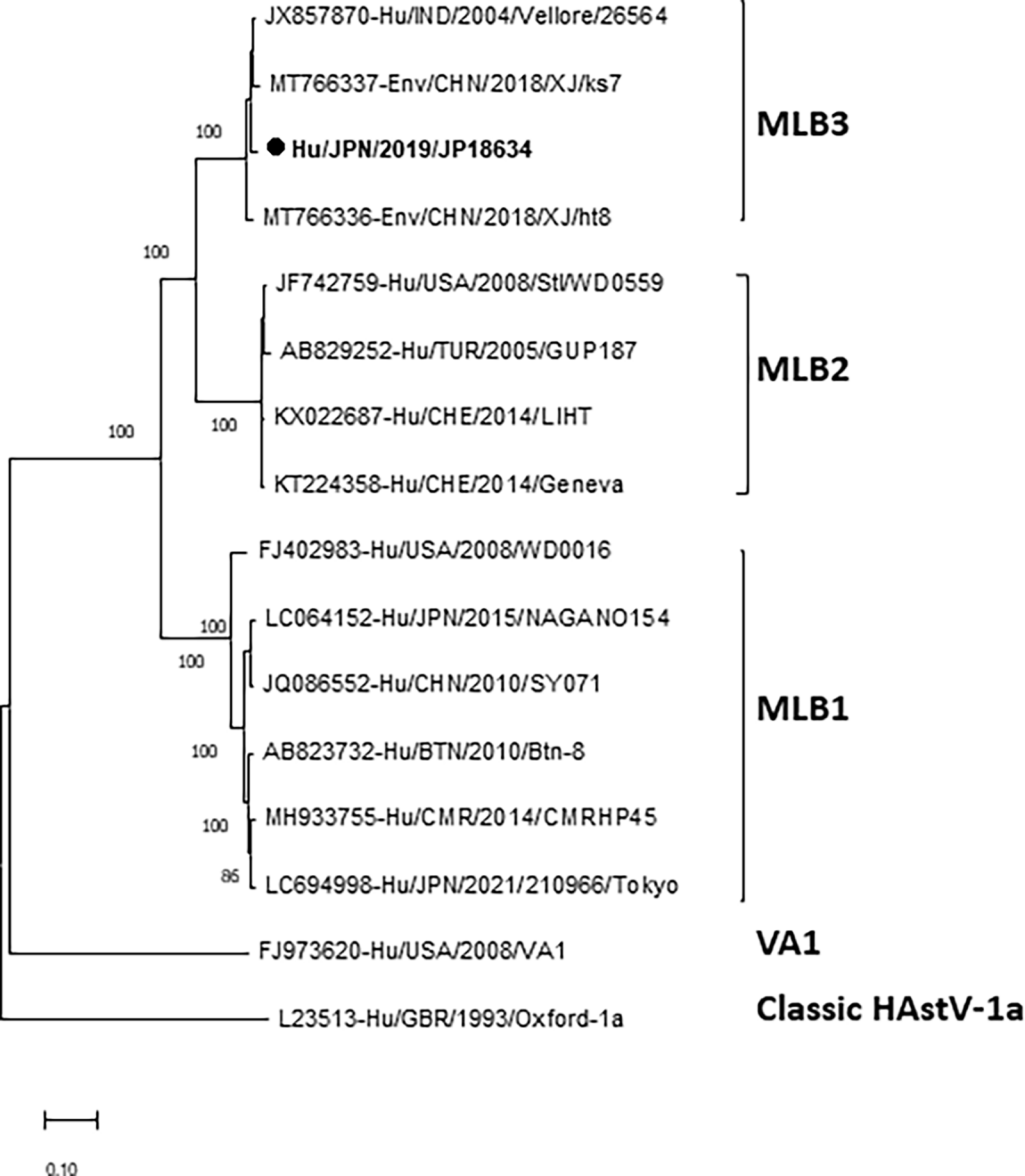
Phylogenetic analysis of nucleotide sequences of the nearly full-length genome (6,684 bp) of MLB3 HAstV strains. Trees were constructed using the maximum-likelihood method based on the Kimura two-parameter model. Scale bar shows nucleotide substitutions per site and bootstrap values of >75 are indicated. HAstV strains detected in this study are shown in bold type. The L23513-Hu/GBR/1993/Oxford-HAstV-1a strain is used as an outgroup.

The rare MLB3 strain (JP18634) was detected for the first time in Japan in this study. The nearly full-length genome nucleotide sequence (6,048 bp) of JP18634 was determined and compared with those of three reference strains, one detected in an Indian child and two from sewage water samples in China, revealing that JP18634 and the other three MLB3 reference strains formed a monophyletic branch in the phylogenetic tree, as shown in Fig. 5. The nucleotide sequence of JP18634 was closely related to that of the human MLB3 Vellore/26564 strain detected in India in 2004, with 97.9% sequence identity. Furthermore, the nucleotide sequence identities with other two sewage MLB3 strains detected in China in 2018 (Env/CHN/2018/XJ/ht8 and Env/CHN/2018/XJ/ks7 strains) were 96.8% and 96.9%, respectively. The nucleotide sequence identities with four MLB2 and six MLB1 strains ranged from only 80.0% to 80.2% and from 73.7% to 73.9%, respectively.

## DISCUSSION

The overall prevalence of HAstV infection among infants and young children with AGE in Japan detected in this study during the 2014–2021 period was 4.6%. The prevalence varied year by year from 1.8% to 7.1%, except for during 2020 to 2021, when HAstV infection was not detected, probably due to the very small number of stool samples collected in 2020 to 2021. The number of AGE patients in Japan dramatically decreased in 2020, probably due to the infection control measures against coronavirus disease 2019 (17, 18). Among the HAstV-infected cases, classic HAstV was detected in 2.0%, which is comparable to the approximately 5.0% rate of classic HAstV infection in children with AGE worldwide (2, 10, 11). It is interesting to point out that among the HAstVs detected in this study, MLB1 was most predominant genotype detected at a rate of 45.4%, whereas classic HAstV1 was detected at 39.2% (Table 1). In addition, MLB2, MLB3, and VA2 were detected at 7.4%, 0.8%, and 3.1%, respectively. Generally, classic HAstV has been reported as by far the most predominant genotype worldwide, accounting for over 50% of cases in all recent reports (2), including two from Thailand (10, 19). So far, one study done by our group has reported a higher prevalence for MLB1 than for classic HAstV1 (16). In that study, specimens were collected in 2012 to 2013 from six different geographic prefectures (Hokkaido, Tokyo, Shizuoka, Kyoto, Osaka, and Saga) in Japan. In the present study, the specimens were collected during the 2014–2021 period from one prefecture (Gunma) in Japan, a different area and time point from that of the previous study, but the results of both studies confirm the other’s findings that MLB1 was the most predominant genotype detected and at higher rate than classic HAstV1 (Table 1). Recently, one study demonstrated that MLB1 and HAstV1 were co-predominant genotypes in Germany from 2018 to 2019 (20), and a previous study conducted by the same group of investigators in 2010 to 2015 indicated that HAstV1 was the predominant genotype compared to MLB1 (21). Further epidemiological study of the prevalence of MLB1 and classic HAstV needs to be continuously monitored and extended to other countries around the world. It has been reported that real-time PCR has higher sensitivity for detecting classic HAstV than conventional PCR (2, 22, 23). In this study, HAstV detection was performed using conventional reverse transcription-PCR (RT-PCR) because this method can detect all HAstVs, including classic, MLB, and VA.

Although various HAstV genotypes have been reported, classic HAstV1 tends to be the predominant genotype worldwide (2, 10, 11, 19, 20, 24, 25), and at least three major lineages (HAstV1a, 1b, and 1d) have been identified (24, 25). In Japan, HAstV1d was reported solely in 2006 to 2007 (26), and HAstV1d together with HAstV1a, with HAstV1d being predominant, was reported in 2008 to 2009 (27). In the present study, conducted in 2014 to 2021, HAstV1a became the only lineage exclusively detected (Fig. 3a). These findings suggest that the lineage of HAstV1 strains circulating in pediatric patients with AGE has changed over time. The alternate circulation of different lineages is regarded as a mechanism of HAstV evolution that reduces selection pressure (25).

We analyzed the full-length genome nucleotide sequences of three HAstV3 strains (JP14641, JP14665, and JP14989) which were the putative recombinant strains. During molecular surveillance screening for HAstVs in Japan and nucleotide sequence analyses of the capsid (ORF2) gene, the full genomes of these HAstV3 strains were identified as belonging to the HAstV3c genotype and were most closely related to HAstV3c strains reported from Italy, Japan, Ireland, the United States, and China (Fig. 4a and b). The HAstV3c PR1365/2012 (KF668570) strain detected in Italy was demonstrated to be the first recombinant strain of HAstV1b/HAstV3c of ORF1b/ORF2 by molecular characterization of the partial nucleotide sequences of ORF1b (28). The data indicated that the three HAstV3 recombinant strains detected in our study and four other strains reported from Japan, the United States, and Ireland were very similar to each other, as shown in Fig. 4b and c, suggesting that these HAstV3 strains are homogenous, stable, and widely distributed worldwide. Several studies have reported various types of HAstV recombinant strains, and the ORF1b/ORF2 junction of HAstV was demonstrated to be the most common recombination breakpoint (29–36). On the other hand, some studies have reported recombinant strains at the ORF1a/ORF1b junction regions or within the ORF2 region (33, 37).

The classic HAstV is one of the leading causes of mild AGE in infants and children worldwide (2). However, the roles of MLB and VA HAstVs in AGE are controversial. A case-control study in Kenya and The Gambia concluded that MLB1 HAstV is associated with diarrhea, while MLB3 HAstV was commonly found in the control subjects and was not associated with diarrhea (38). In contrast, MLB1 HAstV was shown not to be associated with diarrhea in a cohort of Indian children (39). In addition, several studies have demonstrated co-infection of HAstV with other diarrheal viruses in AGE patients (10, 16, 21, 23, 40, 41); the real causative agent needs to be investigated. In the present study, a very high percentage of co-infection of HAstV with other diarrheal viruses was also demonstrated (Table 2). For classic HAstV infection, 60.7% of cases were co-infections with other diarrheal viruses, whereas MLB and VA HAstVs were found co-infecting with other diarrheal viruses at rates of 74.3% and 75.0%, respectively. In these cases, the real causative agent of diarrhea awaits further clarification; in particular, these cases were co-infections with rotavirus A and norovirus GII, which have been proven to be causative agents of acute diarrhea.

The MLB3 strain is a rare genotype. It was reported for the first time in India in 2004, and the complete nucleotide sequence of the strain (Vellore/26564) is available in GenBank (42). In Kenya and The Gambia, MLB3 was reported as the predominant HAstV rather than classic HAstV1 or MLB1 (38). In China, MLB3 strains have been detected in environmental water samples (43). Recently, our group also reported the MLB3 strain in environmental water samples from Japan in 2019 to 2020 (44). Currently, only 3 complete nucleotide sequences, including one Indian and two Chinese strains, are available in GenBank. In the present study, we detected the MLB3 strain for the first time in Japan from a pediatric patient with AGE in 2019 to 2020 (Table 1). We characterized the MLB3 strain (JP18634) detected in this study by performing whole-genome nucleotide sequencing and phylogenetic analyses in comparison with the MLB3 reference strains reported from India and China. Our MLB3 strain is closely related to the Indian and Chinese strains, with nucleotide sequence identities of 97.9% and 96.8% to 96.9%, respectively. The Indian strain was detected in 2004 and the Chinese strains were detected in 2008, whereas our Japanese MLB3 strain was detected from 2019 to 2020. All MLB3 strains detected at different time points and in different countries showed high full-length genome nucleotide sequence identities, ranging from 96.8% to 97.9%, suggesting that they may derive from the same ancestral origin and are relatively stable and circulating worldwide.

In conclusion, this study investigated the prevalence and molecular characterization of HAstVs among infants and children with AGE in Japan over a period of 7 years. The results describe the genetic diversity of HAstV, including the classic, MLB, and VA HAstVs circulating in Japan. A rare MLB3 strain was detected for the first time in Japan in this study. Furthermore, this study reported the detection of recombinant HAstV3 strains in Japan.

## MATERIALS AND METHODS

### Specimen collection.

A total of 2,841 stool samples were collected from children with AGE who visited an outpatient pediatric clinic in Gunma Prefecture, Japan, from July 2014 to June 2021. The 1-year periods designated in this study ran from July to June of the following year. The samples were kept in the freezer at −30°C until use. This study was approved by the research ethical committee of the Nihon University School of Medicine (no. 29-9-0, 29-9-2, and 29-9-3).

### Viral RNA extraction, reverse transcription, and detection of HAstV by PCR.

Viral RNA was extracted from 10% of stool suspension using the QIAamp Viral RNA Minikit (Qiagen, Hilden, Germany) according to the manufacturer’s instructions, and cDNA was prepared using ReverTra Ace transcriptase (Toyobo, Japan) and a random primer (TaKaRa Bio, Shiga, Japan). PCR was then performed with GoTaq polymerase (Promega, Madison, WI) using the forward primer SF0073 (GAYTGGACWCGATTTGATGGTAC) and the reverse primer SF0076 (CTGGCTTAACCCACATTCC) targeting the RNA-dependent RNA polymerase gene that yielded a 409-bp amplicon (16, 45). PCR cycling was performed for 3 min at 94°C, followed by 35 cycles of 94°C for 1 min, 50°C for 1 min, 72°C for 1 min, and a final extension at 72°C for 7 min, and the reaction was then held at 4°C. The PCR products were identified by electrophoresis in 1.5% agarose gel, stained with SYBR Safe DNA stain (Invitrogen, Carlsbad, CA), and visualized under blue LED light. Other diarrheal viruses, including rotaviruses A, B, and C, adenovirus, noroviruses GI and GII, sapovirus, *Aichivirus*, parechovirus A, and enterovirus were also detected using conventional RT-PCR or PCR methods as described previously (46, 47).

### HAstV genotyping of classic, MLB, and VA HAstVs.

The RdRp genotypes of HAstV strains detected in this study were determined by analyzing the RdRp sequences of the PCR products obtained from HAstV screening. In addition, the capsid genotypes were also determined by analyzing capsid nucleotide sequences. The partial RdRp-capsid junction region was also amplified using specific primers for classic, MLB, and VA HAstVs as described previously (34). The PCR for the classic HAstV was first amplified using the forward primer SF0073 and the reverse primer 82b to yield a 1,391-bp amplicon (48), and the unamplified samples were further amplified by semi-nested PCR with specific primer sets (34). PCR cycling was conducted for 3 min at 94°C, followed by 40 cycles of 94°C for 1 min, 50°C for 1 min, 72°C for 1 min, and a final extension at 72°C for 7 min, and the reaction was then held at 4°C. Extension times for PCR products longer than 1,000 bp in size were elongated to 1 min 10 sec or 1 min 40 sec.

### Full genome sequencing of MLB3 and HAstV3 strains.

For amplification of the full-length genome sequences of one MLB3 and three HAstV-3 strains, the PCRs were performed using primers listed in Table 3 and in the previous study (49), respectively. The nucleotide sequences of RdRp, the RdRp-capsid junction region, and the full-length genome were determined by Macrogen, Inc. (Tokyo, Japan).

**TABLE 3:** Primers used for amplification of full-length genome of MLB3

Primer	Sequence (5′→3′)	PCR product size (bp)	Reference
MLB-F01	CCAAGAGTGGTRGKATGGCT	1,236	This study
MLB3-R1236	CTGTGCCACTACAGCCATCA		This study
MLB3-F1062	AAAGACAGTACAGGTAAGGTTGTACAAA	1,246	This study
MLB3-R2307	GHA TATACCCA TGTT GTG CCA		This study
MLB3-F1863	CATAGAATGTTGCGCCGTGG	1,172	This study
MLB3-R3039	CCAACCACATTGTCCAGATG		This study
MLB3-F2730	TRGAGTCTACACCTGCATATC	726	This study
MLB3-R3455	CTGGCAATGTTCTATAAGTGG		This study
SF0073	GAYTGGACWCGATTTGATGGTAC	926	45
AHMLBR1	CAGGYTTAGGCCCAGTTGTA		52
MLB3-F3820	GAGGGGTGGACCAAATGGAG	591	This study
MLB3-R4411	GTTTCACACGGGCCAGAAAC		This study
MLB3-F3912	AACCAGCGAGCTAAACCTCC	1,999	This study
MLB3-R5910	TGCGGCGTATGGTTCTTTCT		This study
MLB3-F5220	AGTGGCAACCATCGTGTCAT	905	This study
MLB3-R6124	CCTCTAAATCTACCTGATTAGA		This study

### Phylogenetic and recombination analyses of HAstV.

The nucleotide sequences were compared with those of the reference strains available in GenBank using Basic Local Alignment Search Tool (BLAST; https://blast.ncbi.nlm.nih.gov/Blast.cgi). Phylogenetic analyses were conducted using MEGA version 7.0.26 software (50, 51). Phylogenetic trees were constructed using the maximum-likelihood method based on the Kimura two-parameter model (for HAstV1 and MLB3 analyses) or the Tamura three-parameter model (for HAstV4 and -V5 and HAstV3 analyses) with 1,000 bootstrap replicates. Lineage designation of the classic HAstV was assigned based on ORF2 nucleotide sequences (33).

### Ethics approval statement.

This study was conducted under the approval of the Ethics Committee of Nihon University School of Medicine (no. 29-9-0, 29-9-1, 29-9-2, and 29-9-3).

### Data availability.

The nucleotide sequences of HAstV strains described in this study have been deposited in GenBank under the following accession numbers: LC731962 to LC732014 and LC732122 to LC732194.
